# *Euphorbia bicolor* Xylene Extract Induces Mitochondrial and Endoplasmic Reticulum Stress-Mediated Apoptotic Pathways in MDA-MB-231 and T47D Cells

**DOI:** 10.3390/ijms27020962

**Published:** 2026-01-18

**Authors:** Mafia Mahabub Rumpa, Nguyen Linh Ngo, Camelia Maier

**Affiliations:** 1 Division of Biology, School of the Sciences, Texas Woman’s University, Denton, TX 76204, USA; mrumpa@twu.edunguyenlngo@gmail.com (N.L.N.); 2 Department of Breast Medical Oncology, MD Anderson Cancer Center, University of Texas, Houston, TX 78712, USA

**Keywords:** breast cancer, resiniferatoxin, *Euphorbia bicolor*, TRPV1, ER-positive, triple-negative, ROS, apoptosis, mitochondrial intrinsic and extrinsic apoptosis, endoplasmic reticulum stress-dependent apoptosis

## Abstract

Breast cancer is a significant cause of death worldwide. Recent research has focused on identifying natural compounds for developing effective cancer treatments. Resiniferatoxin, a transient receptor potential vanilloid 1 (TRPV1) agonist, is a common diterpene in *Euphorbia bicolor* Engelm. & A. Gray (Euphorbiaceae), a plant native to the southern United States that has not been studied before. We investigated the antiproliferative activities and mechanisms of action of *E. bicolor* xylene extract in estrogen receptor-positive T47D and triple-negative MDA-MB-231 cell lines. The extract significantly reduced the viability of T47D and MDA-MB-231 cells in a dose-dependent manner. In MDA-MB-231 cells, the extract induced apoptosis via intracellular calcium overload, triggered by TRPV1 activation. This effect was diminished by the TRPV1 antagonist capsazepine and the calcium chelator BAPTA-AM. Intracellular calcium influx was confirmed through Fura-2 AM staining, revealing that *E. bicolor* phytochemicals activated TRPV1 in MDA-MB-231 cells. Treatment of T47D cells with *E. bicolor* xylene extract resulted in apoptosis associated with reactive oxygen species (ROS) generation (10-fold higher in T47D cells than in MDA-MB-231 cells) and mitochondrial calcium overload. These effects were significantly blocked when cells were pretreated with N-acetyl-l-cysteine (NAC), a ROS inhibitor. Both cell lines underwent apoptosis via multiple mitochondrial- and endoplasmic reticulum stress–mediated pathways. This was supported by the activation of caspases 3, 8, and 9; increased expression of FAS, XBP1s, and CHOP; upregulation of BAX; and downregulation of BCL-2. In addition, PI3K, AKT, and pAKT protein expressions were also reduced in both cell lines, indicating downregulation of PI3K/Akt signaling pathway. Phytochemicals in *E. bicolor* xylene extract could become promising ingredients for developing breast cancer therapeutics.

## 1. Introduction

Breast cancer has emerged as a significant concern for women globally, with 2.26 million reported cases in 2020, surpassing all other forms of cancer and becoming the leading cause of cancer-related deaths in women [1,2]. The number of new cases in the United States had increased by 31% in 2023 [3]. Despite significant advances in cancer diagnosis and treatment, the development of chemotherapeutic agents remains an area of intensive research [4,5]. For centuries, plants have served as a foundation of traditional medicine, with many species containing bioactive compounds that exhibit strong anticancer properties [6]. Plant secondary metabolites with anticancer properties, such as alkaloids, terpenoids, and phenolics, may provide a broad range of therapeutic benefits [7,8,9]. Recent research suggests that phytochemicals could have various therapeutic effects, such as preventing tumor growth by activating several cellular signaling pathways and targeting specific receptors [10,11,12,13]. One of the well-known receptors that can be activated by phytochemicals is the transient receptor potential vanilloid 1 (TRPV1) receptor. TRPV1 receptors are ion channels belonging to the TRP channel superfamily, modulated by several phytochemicals and activating several cell death signaling pathways, thus exhibiting antiproliferative effects [14,15,16,17,18]. The TRPV1 channel is a non-selective cation channel classically associated with nociception and thermosensation [19]. However, accumulating evidence indicates that TRPV1 also participates in diverse physiological and pathological processes, including cancer [14,20,21,22], and is expressed in different carcinoma tissues, including all types of breast cancers [23]. Various plant phytochemicals, including capsaicin, gingerol, piperine, and resiniferatoxin (RTX), are the commonly known activators of the TRPV1 channel [17,20]. In cancer cells, TRPV1 activation leads to an influx of calcium, initiating subsequent signaling cascades and triggering antiproliferative effects [14,24,25]. Further study is required to fully understand the potential of phytochemicals targeting TRPV1 in cancer therapy.

One genus known to have antiproliferative effects is *Euphorbia* (Euphorbiaceae). Many species of *Euphorbia* are used in traditional folk medicine to treat different diseases [26] and have been extensively studied because of their wide range of biological activities, including antiproliferative, anti-inflammatory, and analgesic properties [27,28,29]. Extracts of *Euphorbia* species, such as *E. helioscopia* L., and *E. macroclada* Boiss., applied to different breast cancer cell lines, inhibit cell proliferation through cell cycle arrest and apoptosis and were able to reverse multidrug resistance [30,31,32,33]. *Euphorbia bicolor* Engelm. & A. Gray, also known as Snow-on-the-prairie, is native to south-central USA. No scientific research has been done on this species. Our previous research found that the latex extract of *E. bicolor* and its phytochemicals showed antiproliferative properties in ER-positive MCF-7 and T47D, as well as triple-negative MDA-MB-231 and MDA-MB-469 breast carcinomas, but the mechanisms of action were not determined [32].

The present study aims to determine the antiproliferative mechanisms of action of *E. bicolor* xylene extract on ER-positive T47D and triple-negative MDA-MB-231 cell lines. Resiniferatoxin, a common diterpene present in *E. bicolor* [29], was reported to activate the TRPV1 channel in neurons and cancer cells [34,35]. We hypothesized that *E. bicolor* xylene extract, containing diterpenes, would activate TRPV1 and induce TRPV1-dependent antiproliferative mechanisms of action in the breast cancer cell lines. We report that *E. bicolor* xylene extract possesses antiproliferative properties in both breast cancer cell lines under study and induces apoptosis through multiple cell death pathways. To the best of our knowledge, this is the first study on the antiproliferative mechanisms of action of *E. bicolor* xylene extract on ER-positive T47D and triple-negative MDA-MB-231 breast cancer cell lines.

## 2. Results

### 2.1. Antiproliferative Activity of E. bicolor Extracts in T47D and MDA-MB-231 Cells

**Figure 1:**
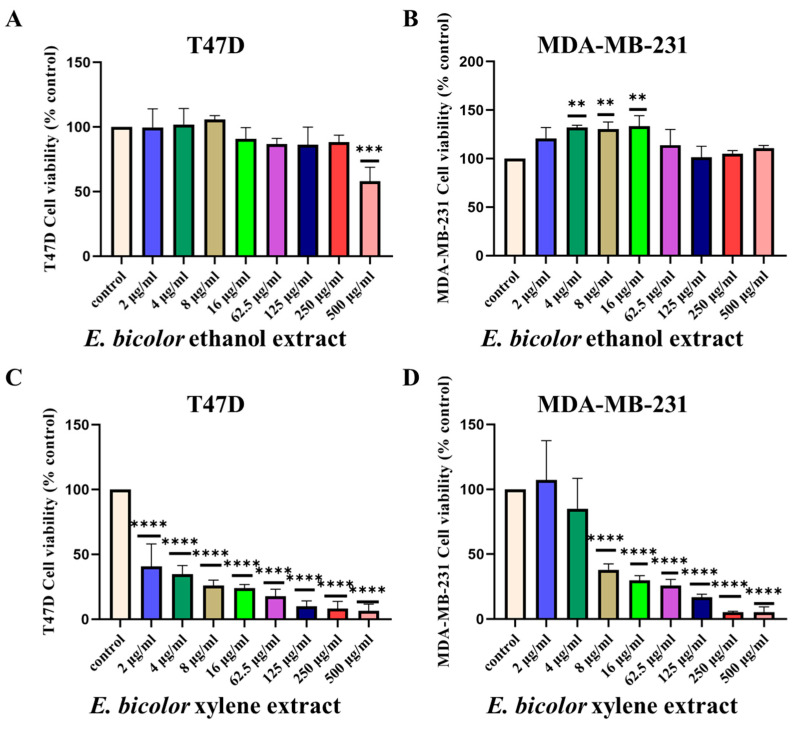

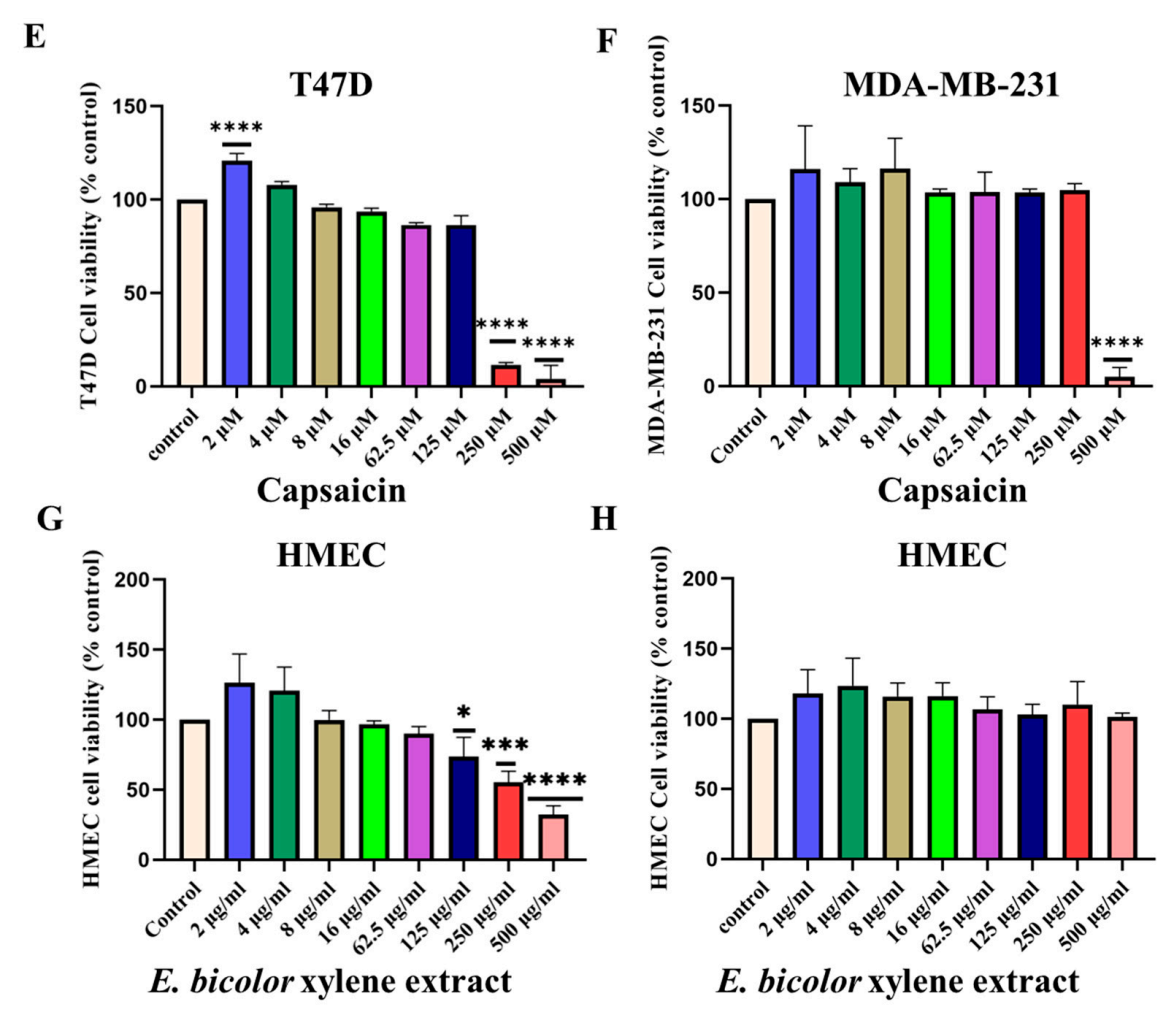
Antiproliferative activities of *E. bicolor* extracts on ER-positive T47D and triple-negative MDA-MB-231 breast cancer cell lines. (**A**,**B**) Effect of *E. bicolor* ethanol extract on T47D and MDA-MB-231 cells; (**C**,**D**) Effect of *E. bicolor* xylene extract on T47D and MDA-MB-231 cells; (**E**,**F**) Effect of capsaicin (+control) on T47D and MDA-MB-231 cell lines; (**G**,**H**) Effect of *E. bicolor* xylene and ethanol extracts on the growth of human mammary epithelial cells (HMECs). Values represent the mean ± SEM of three independent experiments. A one-way ANOVA was performed, followed by Tukey’s post hoc test. * *p* < 0.05, ** *p* < 0.01, *** *p* < 0.001 and **** *p* < 0.0001 vs. DMSO control set.

ER-positive T47D and triple-negative MDA-MB-231 cell lines were treated with increasing concentrations of *E. bicolor* ethanol, xylene extracts, or capsaicin. *E. bicolor* ethanol extract significantly inhibited the cell viability of ER-positive T47D cell lines at 500 µg/mL (Figure 1A). However, *E. bicolor* ethanol extract did not show a reduction in cell viability of triple-negative MDA-MB-231 cells (Figure 1B).

*E. bicolor* xylene extract dose-dependently inhibited the proliferation of ER-positive T47D and triple-negative MDA-MB-231 cell lines (Figure 1C,D). The xylene extract significantly inhibited cell viability, starting at 2 µg/mL in T47D and 8 µg/mL in MDA-MB-231 cell lines. At 500 µg/mL, *E. bicolor* xylene extract significantly inhibited T47D and MDA-MB-231 cell viability by more than 95% (Figure 1C,D). Since the xylene extract is significantly more potent than the ethanol extract in inducing antiproliferative effects, all follow-up experiments were performed with *E. bicolor* xylene extract.

The proliferation of T47D cells was significantly inhibited by capsaicin treatment at 250 µg/mL and 500 µg/mL concentrations (Figure 1E), while MDA-MB-231 cell viability was significantly reduced at 500 µg/mL capsaicin (Figure 1F). In contrast to *E. bicolor* ethanol extract, the xylene extract at higher concentrations (125 µg/mL–500 µg/mL) inhibited the growth of human mammary epithelial cells (HMECs) (Figure 1G,H). Therefore, the rest of the experiments were set up to use 62.5 µg/mL xylene extract to determine the antiproliferative mechanisms.

**Figure 2:**
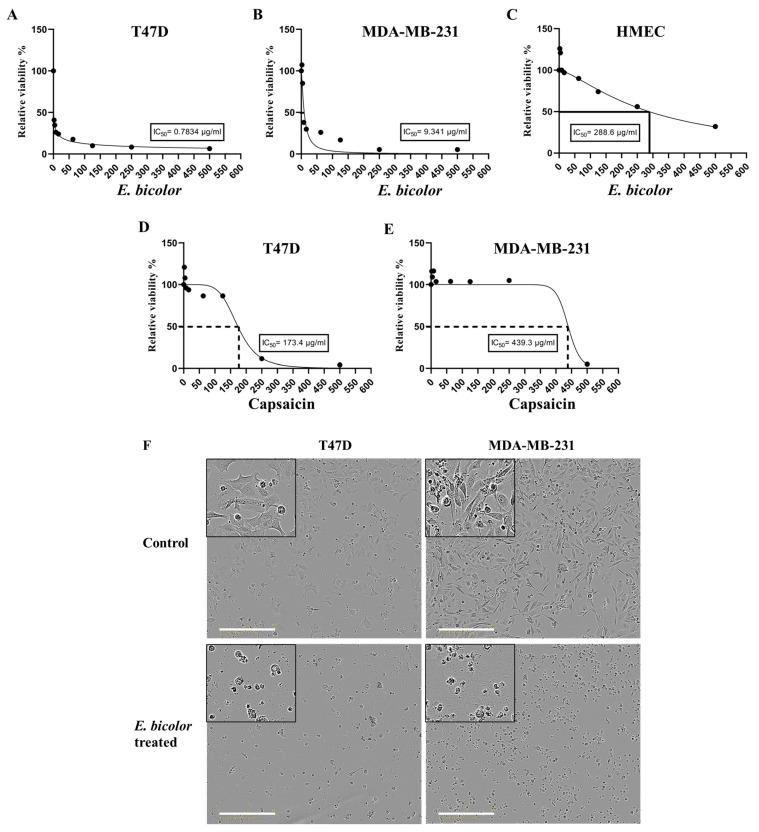
Half-maximal inhibitory concentration (IC50) of *E. bicolor* xylene extract and capsaicin in ER-positive T47D and triple-negative MDA-MB-231 breast cancer cell lines. (**A**) T47-D; (**B**) MDA-MB-231; (**C**) HMEC; (**D**,**E**) IC50 of capsaicin in T47D and MDA-MB-231 cell lines, respectively. (**F**) T47D and MDA-MB-231 cell morphology 72 h after *E. bicolor* xylene extract treatment (62.5 μg/mL) observed using the live cell imaging system IncuCyte. Representative pictures of cell morphologies three days after treatment. Scale bar: 400 μm.

The half-maximal inhibitory concentration (IC50) of *E. bicolor* xylene extract for T47D cells was 0.7834 µg/mL (Figure 2A), and for MDA-MB-231 cells was 9.341 µg/mL (Figure 2B). The IC50 of *E. bicolor* xylene extract for HMECs was 288.6 µg/mL (Figure 2C). The IC50 of capsaicin for T47D cells was 173.4 µg/mL (Figure 2D), and MDA-MB-231 cells were 439.3 µg/mL (Figure 2E).

Three days after *E. bicolor* xylene extract treatment (62.5 μg/mL), fewer cells as well as cell morphological changes were observed in both cell lines. Cells were smaller and more spherical, partially or completely detached from the bottom of the wells after treatment, indicative of the cytotoxic effects of *E. bicolor* xylene extract (Figure 2F).

### 2.2. E. bicolor Xylene Extract Induces Apoptosis in T47D and MDA-MB-231 Cells

**Figure 3:**
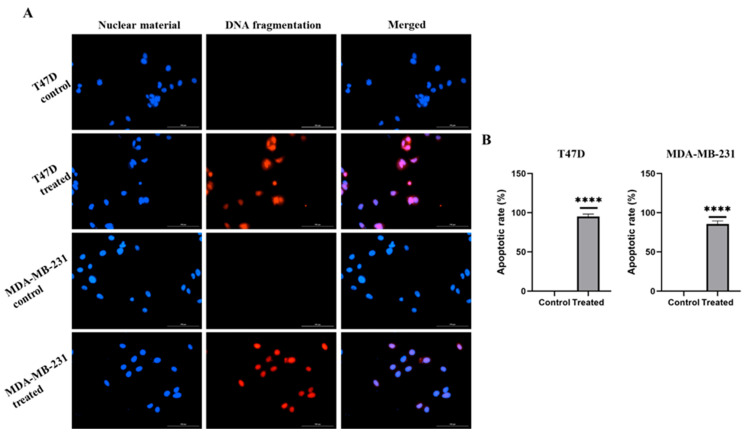
Apoptosis detected by TUNEL assay. (**A**) T47D and MDA-MB-231 cells treated with *E. bicolor* xylene extract. Red fluorescence indicates that the modified dUTPs from the TUNEL test kit bind to the apoptotic broken DNA. The blue fluorescence of Hoechst 33342 indicates nuclear material. Cells were visualized with LionHeart FX microscope, scale bar: 100 μm (**B**) Apoptotic rate (%) of TUNEL assays. The number of apoptotic cells was counted using LionHeart BioTek Gen5.0 software. Unpaired Welch’s *t*-test, **** *p* < 0.0001 vs. DMSO control set.

T47D and MDA-MB-231 cells were treated with *E. bicolor* xylene extract (62.5 µg/mL) for 24 h, and TUNEL assays were performed to detect apoptosis. Typical DNA fragmentation in T47D and MDA-MB-231 cell lines was observed (Figure 3A). The number of apoptotic T47D and MDA-MB-231 cells significantly increased with *E. bicolor* xylene extract treatments, as indicated by the relative red fluorescence intensity of Alexa 594 (Figure 3B).

### 2.3. ROS Production Activated E. bicolor-Induced Apoptosis in T47D Cells

**Figure 4:**
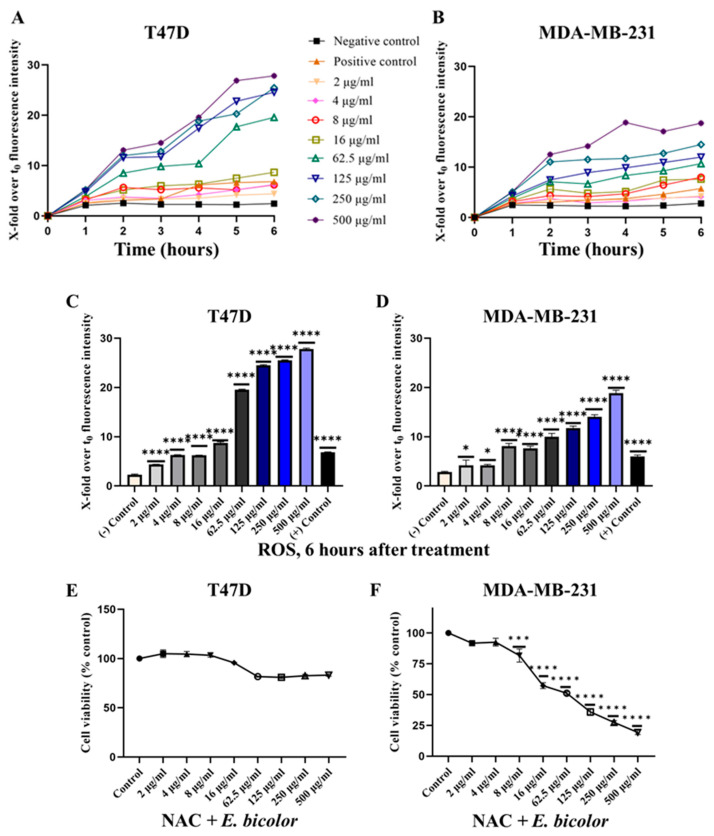
ROS level detection after *E. bicolor* xylene extract treatment. (**A**,**B**) Increased ROS levels with time in T47D and MDA-MB-231 cells. (**C**,**D**) ROS accumulation at 6 h of treatment. Tert-butyl hydroperoxide (TBHP, 250 μM) was used as the positive control, and DMSO (0.1%) as the negative control. (**E**,**F**) Cell viability after inhibition of ROS formation with NAC in T47D and MDA-MB-231 cells. Cells were pretreated with NAC (5 mM), a ROS inhibitor, for 1 h, then treated with or without *E. bicolor* xylene extract for 72 h. Cell viability was measured using MTS assay. Values represent the mean ± SEM of three independent experiments. One-way ANOVA, * *p* < 0.05, *** *p* < 0.001, **** *p* < 0.0001 vs. DMSO control set.

T47D and MDA-MB-231 cells were treated with *E. bicolor* xylene extract, and reactive oxygen species (ROS) production was monitored using 2′,7′-dichlorofluorescin diacetate (DCFDA) to investigate whether *E. bicolor* treatment could generate ROS accumulation in cells. The DCFDA fluorescence intensity increased in a dose-dependent manner with increasing time of *E. bicolor* xylene extract treatment in both T47D and MDA-MB-231 cells (Figure 4A,B). *E. bicolor* xylene extract treatment significantly and dose-dependently triggered intracellular ROS accumulation in T47D and MDA-MB-231 cells compared to the negative control and 20× and 30× higher than the positive control (Figure 4C,D). At higher doses of *E. bicolor* xylene extract treatments, intracellular ROS accumulation was found to be ten times greater in T47D cells than in MDA-MB-231 cells (Figure 4C). To determine whether ROS generation was associated with *E. bicolor*-induced apoptosis, T47D and MDA-MB-231 cells were pretreated with the ROS inhibitor N-Acetyl-L-cysteine (NAC) for 1 h and then treated with *E. bicolor* xylene extract. Pretreatment with NAC significantly ameliorated the effect of *E. bicolor* in T47D cells (Figure 4E), suggesting that ROS generation is involved in *E. bicolor* diterpene extract-induced apoptosis in T47D cells. However, NAC could not inhibit the effect of *E. bicolor* xylene extracts in MDA-MB-231 cells (Figure 4F), suggesting that *E. bicolor* extracts induced apoptosis in a TRPV1-dependent manner and only partially through ROS generation.

### 2.4. Blocking TRPV1 and Chelating Calcium Could Not Completely Inhibit the Antiproliferative Activity of E. bicolor Extract in T47D

**Figure 5:**
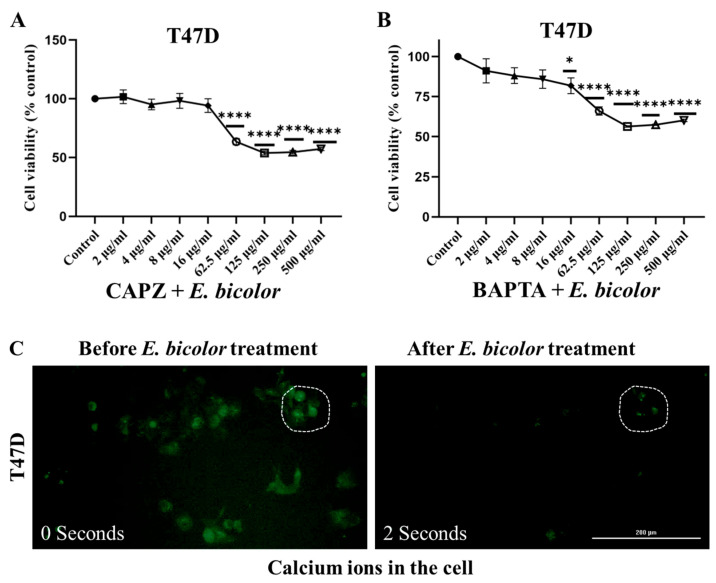

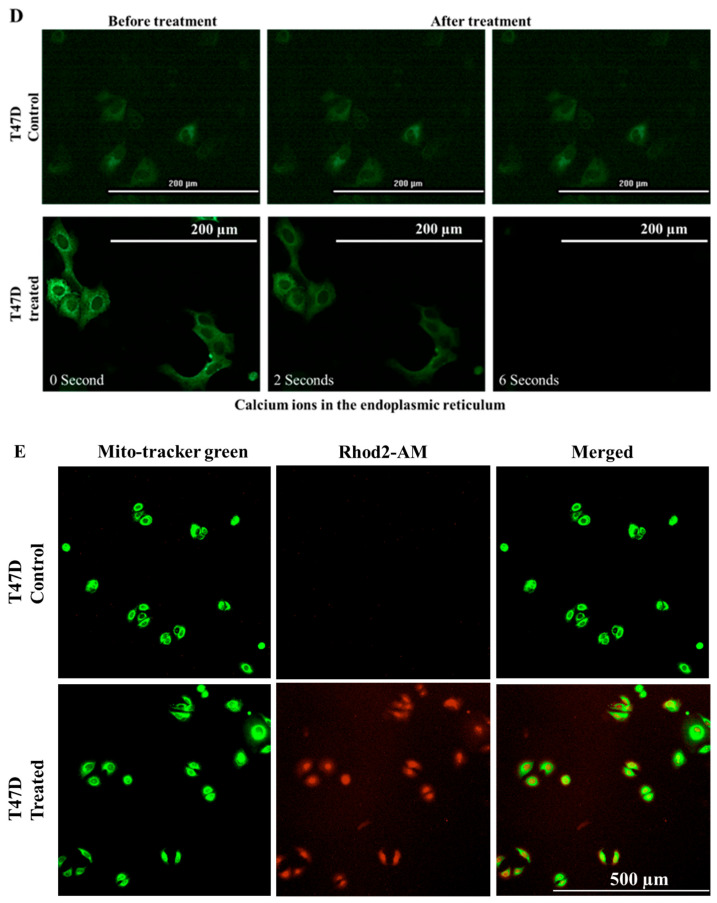
Calcium dynamics in T47D induced by *E. bicolor* xylene extract treatment. (**A**) Cell viability after blocking TRPV1 with capsazepine (CAPZ), a TRPV1 antagonist. Cells were pretreated with 10 μM CAPZ before treatment with *E. bicolor* xylene extract. (**B**) Cell viability after chelating calcium with BAPTA-AM. Cells were pretreated with 1 μM of BAPTA-AM, and then treated with *E. bicolor* extract (62.5 μg/mL). MTS assays were employed for measuring cell viability. Values represent the mean ± SEM of three independent experiments. One-way ANOVA, * *p* < 0.05, **** *p* < 0.0001 vs. DMSO control set. (**C**) Visualization of calcium dynamics in T47D cells. Cells were treated with 5 μM of Fura2-AM and then with 62.5 μg/mL of *E. bicolor* xylene extract in HBSS buffer (with Ca^2+^ and Mg^2+^). Fluorescent images were captured every 2 s for 10 min. Dotted circles on the representative Ca^2+^ fluorescence images represent regions of interest. (**D**) Visualization of ER calcium dynamics with a fluorescent reporter for ER calcium signaling in GCaMP6-210 plasmid variant. T47D cells were grown in a 24-well plate for 24 h and transfected with GCaMP6-210 plasmid variant. Twelve hours after incubation, cells were exposed to 62.5 μg/mL of *E. bicolor* in HBSS buffer (with Ca^2+^ and Mg^2+^). The fluorescent images were captured every 2 s and recorded for 10 min. (**E**) visualization of mitochondrial calcium with MitoTracker Green. T47D cells were simultaneously loaded with 10 μM of MitoTracker Green and 5 μM of Rhod2-AM and then exposed to 62.5 μg/mL of *E. bicolor* xylene extract. A LionHeart FX microscope, 4× magnification, monitored the corresponding fluorescence signal.

Resiniferatoxin, commonly known as a TRPV1 agonist, is present in *E. bicolor* [29,34]. Therefore, we hypothesized that TRPV1 would be activated by *E. bicolor* xylene extract, causing an influx of calcium that would lead to cell death. The T47D cells were treated with *E. bicolor* xylene extract after pretreating them with 10 μM of capsazepine (CAPZ), a TRPV1 antagonist. TRPV1 inhibition increased the cell proliferation only at *E. bicolor* extract concentrations of 2–16 µg/mL. Capsazepine could not completely block the effect of higher concentrations of *E. bicolor* xylene extract (Figure 5A). To determine the calcium involvement in apoptosis, 1 μM of the calcium chelator BAPTA-AM was used to pretreat T47D cells, after which they were treated with *E. bicolor* xylene extract. Chelating calcium increased the cell proliferation only at concentrations of 2–8 µg/mL of *E. bicolor* extract. Chelating calcium could not completely block the effect of higher concentrations of *E. bicolor* extract (Figure 5B). Fura2-AM staining was employed to check intracellular calcium concentrations and activation of TRPV1. Fluorescence followed a downward trend immediately after the *E. bicolor* treatment (Figure 5C), revealing that TRPV1 may not be involved in the antiproliferative mechanism of action.

In search of calcium-regulated apoptotic mechanism of action, an endoplasmic reticulum-targeted low-affinity GCaMP6-210 plasmid variant, a fluorescent reporter for ER calcium signaling, was used to visualize ER Ca^2+^ dynamics. T47D cells were transfected with the GCaMP6-210 variant, and immediately after *E. bicolor* xylene extract treatment, calcium concentration (green fluorescence) was observed. GCaMP6-210 fluorescence followed a downward trend (Figure 5D), suggesting that *E. bicolor* treatment in T47D cells triggers the release of calcium from ER. This suggests that TRPV1 might not be involved in the cell death of *E. bicolor*-treated T47D cells.

Rhod2-AM was used to monitor mitochondrial calcium. Twenty-seven seconds after *E. bicolor* xylene extract treatment, Rhod2-AM was localized within the mitochondria of T47D cells (Figure 5E).

### 2.5. TRPV1 Activation Triggered E. bicolor-Induced Apoptosis in MDA-MB-231 Cells

**Figure 6:**
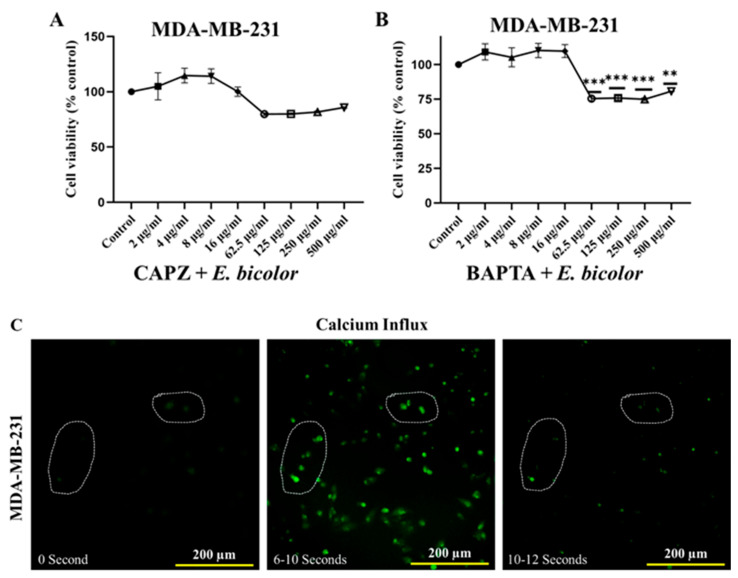

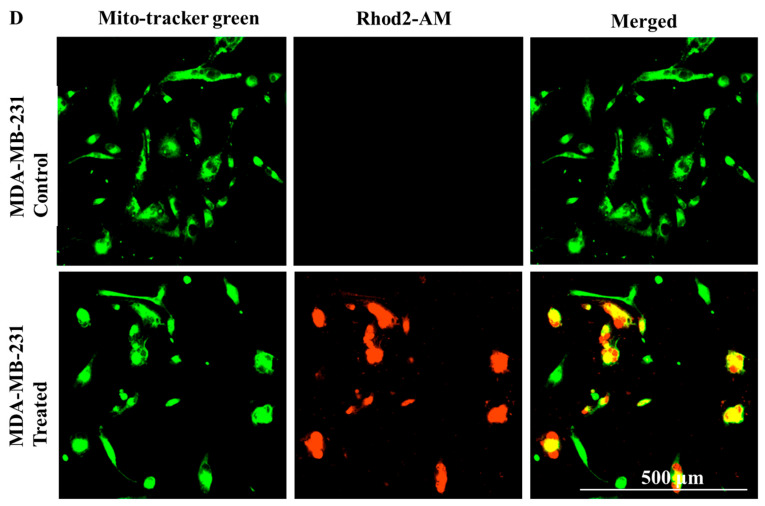
Calcium dynamics in MDA-MB-231 cells induced by *E. bicolor* xylene extract. (**A**) Cell viability after blocking TRPV1 with CAPZ. Cells were pretreated with 10 μM CAPZ before being treated with *E. bicolor* extract (62.5 μg/mL). (**B**) Cell viability after chelating calcium with BAPTA-AM. Cells were pretreated with 1 μM of BAPTA-AM and then treated with *E. bicolor* xylene extract (62.5 μg/mL). MTS assay was used for cell viability. Values represent the mean ± SEM of three independent experiments. One-way ANOVA, ** *p* < 0.01, *** *p* < 0.001 vs. DMSO control set. (**C**) Visualization of calcium dynamics in MDA-MB-231 cells. Before treatment with *E. bicolor* xylene extract (62.5 μg/mL) in HBSS buffer (with Ca^2+^ and Mg^2+^), cells were loaded with 5 μM of Ca^2+^ sensitive fluorescent probe Fura2-AM. Fluorescent images were captured every 2 s. Representative Ca^2+^ fluorescence images at indicated time points are shown, and regions of interest were drawn with dotted circles. (**D**) Visualization of mitochondrial calcium with MitoTracker Green. Cells were simultaneously loaded with 10 μM of MitoTracker Green and 5 μM of Rhod2-AM and then treated with *E. bicolor* extract (62.5 μg/mL). LionHeart FX microscope (4× magnification) was used to monitor fluorescence signals.

MDA-MB-231 cells were pretreated with 10 μM of CAPZ and treated with *E. bicolor* xylene extract. TRPV1 inactivation by CAPZ significantly increased the cell viability of extract-treated cells (Figure 6A), indicating that *E. bicolor* extract reduces the viability of MDA-MB-231 cells in a TRPV1-dependent manner. To see the effect of calcium chelation, which could oppose the above scenario and increase cell viability, MDA-MB-231 cells were exposed to *E. bicolor* xylene extract after pretreatment with 1 μM of the calcium chelator BAPTA-AM. At low extract concentrations (2–16 μg/mL), chelating calcium with BAPTA-AM increases the cell viability by blocking the effect of *E. bicolor* extract (Figure 6B). However, at higher extract concentrations (62.5–500 μg/mL), MDA-MB-231 cells exhibited a significant decrease in viability, likely attributable to the cytotoxic effects of *E. bicolor* xylene extract at elevated doses.

To check the calcium dynamics induced by the activation of TRPV1, Fura2-AM staining was used and an increase in intracellular calcium was observed. Fluorescence intensity followed an upward trend immediately after the *E. bicolor* xylene extract treatment, which lasted for 6–10 s (Figure 6C). To determine if this activation could lead to the accumulation of calcium in mitochondria, Rhod2-AM was used to monitor mitochondrial calcium. An immediate accumulation of calcium in the mitochondria was observed. Within 8 s after *E. bicolor* xylene extract treatment, Rhod2-AM was predominantly localized in the mitochondria of MDA-MB-231 cells (Figure 6D).

### 2.6. E. bicolor Xylene Extract Induced Mitochondrial Intrinsic and Extrinsic Apoptotic Signaling Pathways in T47D and MDA-MB-231 Cells

**Figure 7:**
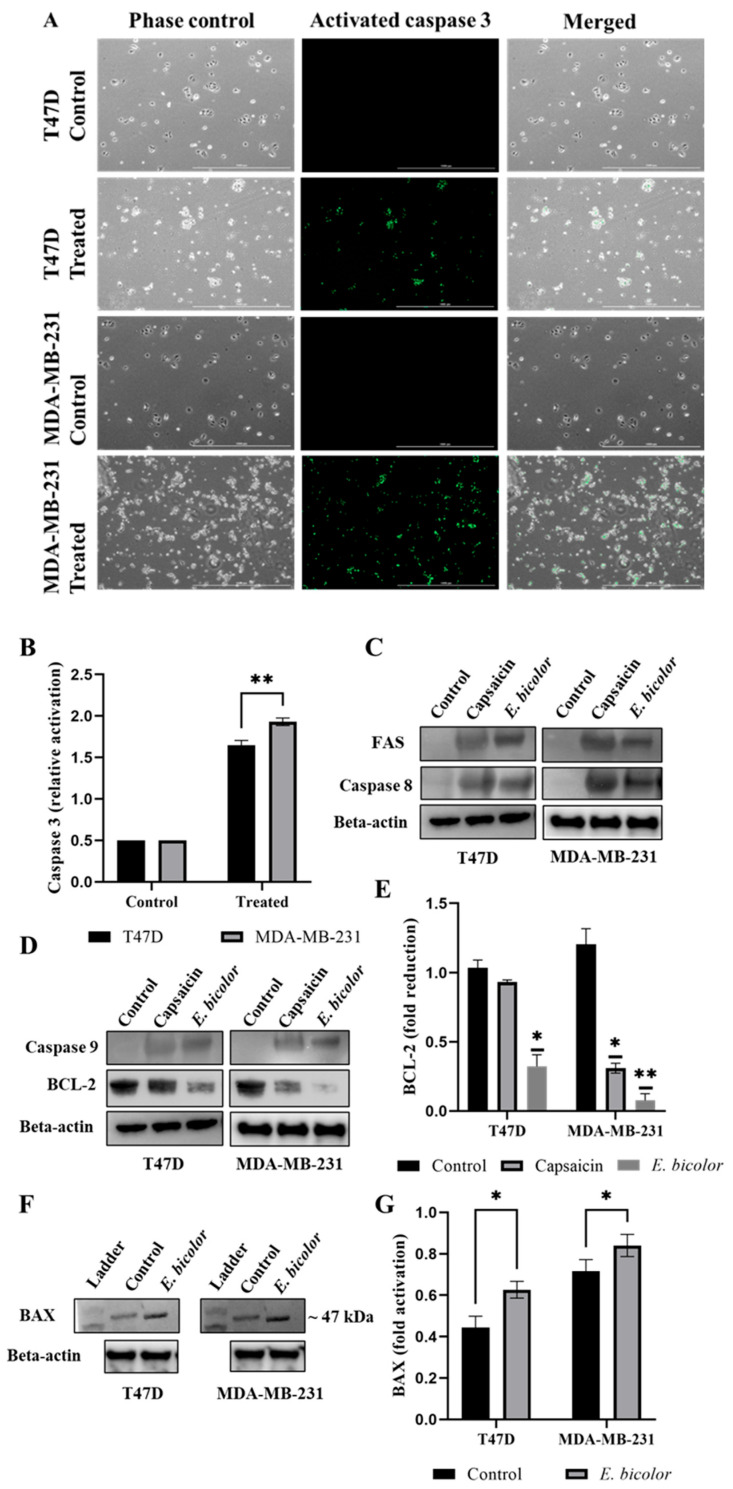
*E. bicolor* xylene extract induces mitochondrial intrinsic and extrinsic apoptosis in T47D and MDA-MB-231 cells. (**A**) Visualization of activated caspase 3 after *E. bicolor* xylene extract treatment (62.5 µg/mL) with a LionHeart FX microscope; scale bar, 200 μm. (**B**) Relative activation of caspase 3 measured with LionHeart BioTek Gen5.0 software. Two-way ANOVA, ** *p* < 0.001. (**C**) Western blot analyses of mitochondrial extrinsic apoptotic proteins FAS and caspase 8. T47D and MDA-MB-231 cells were treated with *E. bicolor* (62.5 µg/mL) or capsaicin (500 µM) for 24 h; cell lysates were isolated and immunoblot analyses of proteins FAS and caspase 8 were performed. (**D**) Western blot analyses of mitochondrial intrinsic apoptotic protein caspase 9 and antiapoptotic BCL-2. T47D and MDA-MB-231 cells were treated with *E. bicolor* extract (62.5 µg/mL) or capsaicin (500 µM) for 24 h, and cell lysates were isolated, and immunoblot analyses of proteins caspase 9 and BCL-2 were performed. (**E**) Fold reduction in BCL-2. One-way ANOVA, * *p* < 0.05, ** *p* < 0.01. (**F**) Western blot analyses of proapoptotic BAX protein. (**G**) Fold activation of BAX. Unpaired *t*-test, * *p* < 0.05. Representative Western blots, beta-actin served as loading control. Control, non-treated cells (DMSO).

Mitochondria are critical mediators of apoptotic signaling pathways. Activated/cleaved caspase 3 (green fluorescence) was detected in both T47D and MDA-MB-231 cells treated with *E. bicolor* xylene extract (62.5 μg/mL). Activation of caspase 3 was significantly higher in MDA-MB-231 cells compared with T47D cells (Figure 7A,B). Capsaicin (positive control) and *E. bicolor* extract treatments were also associated with the expression of caspase 8 and FAS, indicating induction of mitochondrial extrinsic apoptosis (Figure 7C). Capsaicin and *E. bicolor* xylene extract treatments led to the expression of caspase 9 and reduction in anti-apoptotic BCL-2 protein compared to the DMSO control (Figure 7D). However, the expression of BCL-2 was significantly lower in *E. bicolor* extract-treated cells than in control and capsaicin (Figure 7E). The proapoptotic BAX protein showed a higher molecular weight band (47 KDa) in capsaicin and *E. bicolor* extract-treated cells, compared to its known average molecular weight (21 KDa) (Figure 7F). The fold activation of BAX is significantly higher than that of DMSO control (Figure 7G), indicating that *E. bicolor* xylene extract treatment also led to mitochondrial intrinsic apoptotic pathway.

### 2.7. E. bicolor Xylene Extract Downregulates the PI3K/AKT Signaling Pathway in T47D and MDA-MB-231 Cells

**Figure 8:**
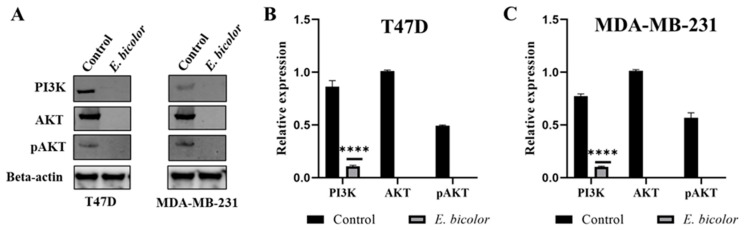
*E. bicolor* xylene extract inhibits the PI3K/AKT signaling pathway in T47D and MDA-MB-231 cells. (**A**) Western blot analyses after *E. bicolor* extract treatment (62.5 µg/mL) for 24 h. Representative Western blots, control—untreated cells (DMSO); beta-actin was loading control. (**B**,**C**) Relative expressions of PI3K, AKT, and pAKT in T47D and MDA-MB-231 cells, respectively. Unpaired *t*-test, **** *p* < 0.0001 vs. DMSO control set. Control, untreated cells (DMSO).

The protein kinase B (AKT) plays essential roles in cell survival, growth, and proliferation by regulating cellular signaling [36]. To check for the inhibition of the PI3K/AKT signaling pathway as one of the antiproliferative mechanisms of action of *E. bicolor* xylene extract, expression of PI3K, phosphorylated AKT, and non-phosphorylated proteins was evaluated. Reduced or no expression of PI3K, AKT, and pAKT proteins was observed in both *E. bicolor* extract-treated T47D and MDA-MB-231 cell lines (Figure 8), suggesting inhibition of the PI3K/AKT signaling pathway by *E. bicolor* xylene extract.

### 2.8. E. bicolor Xylene Extract Induces ER-Dependent Apoptosis in T47D and MDA-MB-231 Cells

**Figure 9:**
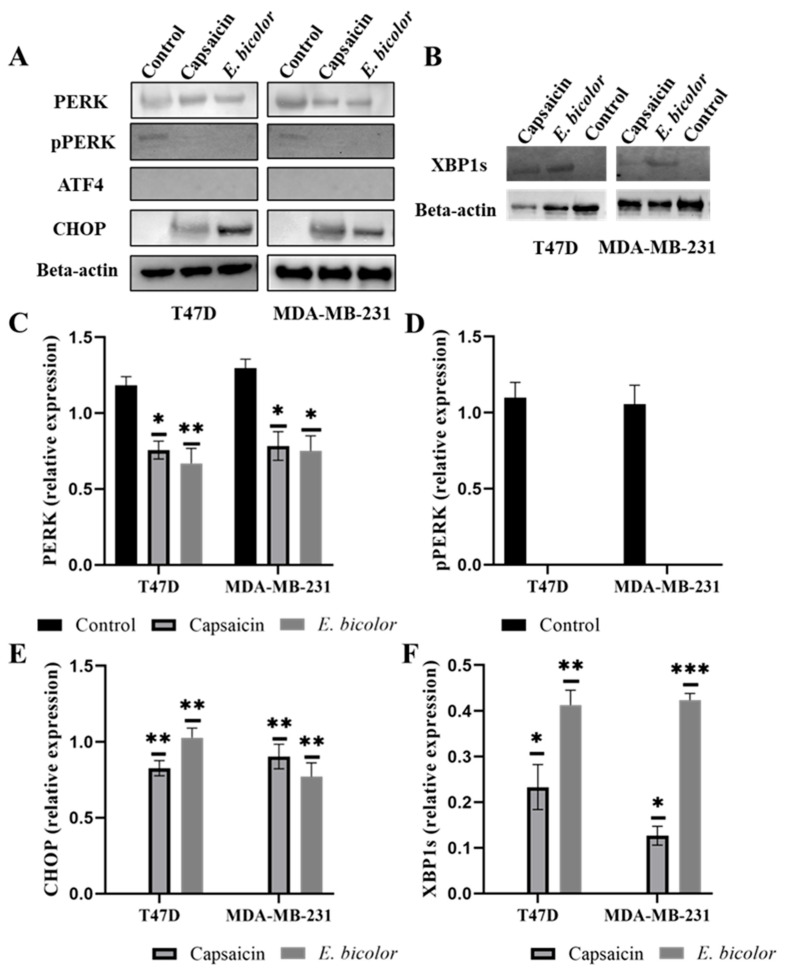
*E. bicolor* xylene extract induces ER stress-dependent apoptosis in T47D and MDA-MB-231 cells. (**A**,**B**) Western blot analysis of ER-stress mediated apoptotic proteins in T47D and MDA-MB-231 cells treated with *E. bicolor* (62.5 µg/mL) or capsaicin (500 µM) for 24 h. Representative Western blots, beta-actin was loading control. (**C**–**E**) Relative expression of PERK, pPERK, and CHOP in T47D and MDA-MB-231 cells. (**F**) Relative expression of XBP1s. One-way ANOVA, * *p* < 0.05, ** *p* < 0.01, *** *p* < 0.0001 vs. DMSO control set. Control, cells in DMSO medium. ImageJ software was used to determine fold protein expression.

Disruption of cytosolic calcium homeostasis and generation of excessive ROS induce ER stress [37]. To assess the effect of *E. bicolor* xylene extract on ER stress-dependent apoptotic protein kinase RNA-like ER kinase (PERK)—activating transcription factor 4 (ATF4)—C/EBP homologous protein (CHOP) (PERK/ATF4/CHOP) and activating transcription factor 6 (ATF6)—spliced X-box binding protein 1—C/EBP homologous protein (CHOP) (ATF6/XBP1s/CHOP) pathways was further investigated. *E. bicolor* extract treatment led to the expression of PERK and CHOP (endoplasmic apoptotic protein) (Figure 9A). However, expression of phosphorylated PERK and ATF4 proteins was absent in capsaicin and *E. bicolor* xylene extract-treated T47D and MDA-MB-231 cells (Figure 9A), indicating the absence of PERK/ATF4/CHOP pathway. We further checked the expression of XBP1s protein, which activates CHOP and can be induced by ER stress leading to the ER-stress mediated apoptotic ATF6/XBP1s/CHOP pathway. Expression of XBP1s was found in both capsaicin and *E. bicolor* extract-treated cells, suggesting the presence of the ATF6/XBP1s/CHOP pathway.

## 3. Discussion

**Figure 10:**
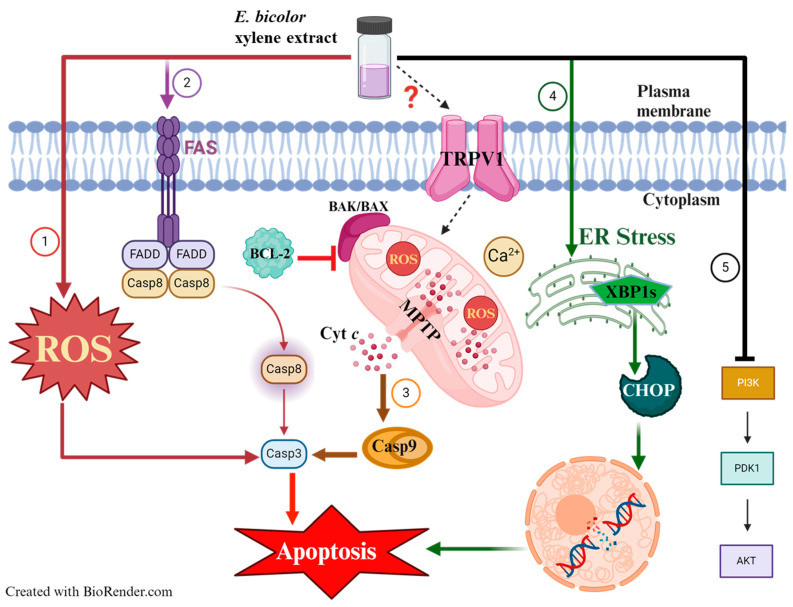
Proposed model of the mechanisms of action of *E. bicolor* xylene extract on ER-positive T47D breast cancer cells. (1) Plant extract increases ROS level. The elevated ROS level could trigger several apoptotic pathways. Increasing levels of ROS led to caspase 3 activation, increasing proapoptotic proteins BAK/BAX, decreasing anti-apoptotic BCL-2, and leading to mitochondrial intrinsic (3) and extrinsic (2) apoptotic pathways by activating FAS, caspase 8, and caspase 3. It may be that plant extract activates the FAS-caspase 8 pathway directly (2). (4) ER-stress mediated apoptosis pathway. Increasing ROS level and/or *E. bicolor* extract led to ER stress. ER-stress activates XBP1s, which activate CHOP, resulting in XBP1s-CHOP-mediated apoptosis. (5) *E. bicolor* xylene extract downregulates the PI3K-AKT signaling pathway. The question mark (?) signifies that TRPV1 may or may not be involved in triggering apoptotic pathways, depending on *E. bicolor* xylene extract concentrations. Created with BioRender.com.

**Figure 11:**
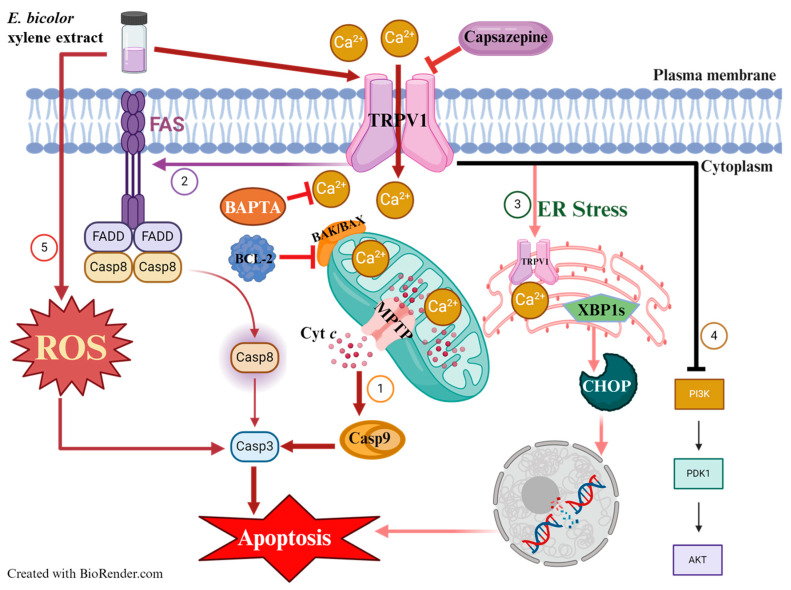
Proposed model of the mechanisms of action of *E. bicolor* xylene extract on triple-negative MDA-MB-231 breast cancer cells. TRPV1 is activated by plant extract, resulting in calcium influx into cells. The increased intracellular calcium levels contribute to several apoptotic pathways, such as the mitochondria-mediated intrinsic, extrinsic, and ER-stress mediated pathways. (1) Mitochondrial membrane potential goes through a transient depolarization due to accumulation of Ca^2+^ in mitochondria, resulting in opening of the mitochondrial permeability transition pores (MPTPs) and release of cytochrome c (cyt c). As a result, caspases are activated, increasing expression of proapoptotic proteins BAK/BAX, decreasing anti-apoptotic BCL-2, thus inducing apoptosis. (2) Calcium influx through TRPV1 triggers the extrinsic pathway by activating FAS, caspase 8, and caspase 3, contributing to apoptosis. (3) TRPV1 activation at the plasma membrane resulting in calcium influx also leads to ER stress. TRPV1 in the ER membrane is another source of calcium release into the cell. ER stress activates XBP1s, which activates CHOP, leading to XBP1s-CHOP-mediated apoptosis. (4) *E. bicolor* xylene extract downregulates the PI3K-AKT signaling pathway. (5) Plant extract increases cellular ROS levels triggering several apoptotic pathways. Created with BioRender.com.

Worldwide, breast cancer is the most prevalent cancer diagnosed in women [38]. Expanding advances in diagnosis is essential to reduce the global mortality from breast cancer. In this context, numerous plant species contain natural compounds with demonstrated anticancer potential [39]. Conducting focused research on bioactive plant chemicals, scientists could develop novel therapeutic options for breast cancer treatment. The present study tested the antiproliferative activities of *E. bicolor* xylene extract on ER-positive T47D and triple-negative MDA-MB-231 breast cancer cell lines and found that the extract significantly reduced the proliferation of both breast cancer cell lines (Figure 1, Figure 2 and Figure 3). Previously, it was reported that *E. bicolor* latex ethanol extract possesses antiproliferative activity on T47D and MDA-MB-231 breast cancer cell lines [32], but no mechanisms of action were provided. This study proposes the mechanisms of action of *E. bicolor* diterpene extract in the two cell lines under study presented in Figure 10 and Figure 11.

Plants of *Euphorbia* genus are well-known for their antiproliferative effects. *E. hirta* L. methanolic extract suppressed MCF-7 cell growth at 24 h with a GI50 value of 25.26 µg/mL [40]. A study on the effect of *E. macroclada* acetone extract made from leaves and flowers reported significant cytotoxicity in MCF-7 breast cancer cells [41]. *E. macroclada* tissues extracted with dichloromethane and ethyl acetate showed cytotoxic effects on MDA-MB-468 cells. In contrast, the methanol and latex extracts in DMSO did not exhibit cytotoxic effects [33]. Another study on the biological activity of *E. tirucalli* L. stem extracts in methanol, butanol, and hexane resulted in inhibition of MCF-7 and MDA-MB-231 cell proliferation in a concentration-dependent manner [42]. Our study found that *E. bicolor* ethanol extract possesses antiproliferative effects only at higher concentrations in ER-positive T47D cells. However, no antiproliferative effect was observed on the triple-negative MDA-MB-231 cells (Figure 1). Different solvent extracts of *Euphorbia* species, as well as extract concentration used, show a wide range of antiproliferative effects in breast cancer cell lines. In our study, we do not claim that diterpenes are the exclusive constituents of the xylene extract, nor do we assign the observed biological effects to a single compound. The results are interpreted as arising from an extract whose activity reflects the combined effects of its components.

Compared to *E. bicolor* ethanol extract, the xylene extract was toxic to HMEC at high concentrations. Previous research showed that the diterpene RTX was not toxic to normal cells [32,43,44]. However, *E. bicolor* xylene extract contains other chemicals besides the diterpene RTX. Biochemical identification results from our lab, previously published, showed that *E. bicolor* latex extract contains the diterpenes RTX and abietic acid [29]. The HMEC cytotoxicity may be a combined effect of RTX, abietic acid, and other chemicals in the extract. Therefore, future research should focus on chemical identification and isolation of other diterpenes in *E. bicolor* extracts, to individually be used to study antiproliferative effects, thus selecting the nontoxic ones for possible drug development.

In our attempt to understand the antiproliferative mechanisms of action of *E. bicolor* xylene extract in T47D and MDA-MB-231 cells, we took into consideration the activation of TRPV1, calcium influx, and apoptotic markers. Calcium ions as secondary messengers have important regulatory functions, also being involved in cellular processes of cancer development [43,44]. TRPV superfamily of plasma membrane ion channels is one of the most active calcium-permeable superfamilies in regulating Ca^2+^ influx [14,25]. It is known that capsaicin activates TRPV1 in cancer cells and has potent anticancer activity against certain cancer types through both Ca^2+^-dependent and -independent mechanisms [44,45]. In our study, capsaicin showed antiproliferative activity in T47D and MDA-MB-231 cells at much higher concentrations than *E. bicolor* xylene extract, which may be because the extract contains various diterpenes, and they may synergistically work to induce the observed antiproliferative effects.

Blocking TRPV1 and chelating calcium increased the T47D and MDA-MB-231 cell viability but did not completely counteract the effect of *E. bicolor* xylene extract. On the other hand, inhibiting ROS formation with NAC completely blocked the effect of *E. bicolor* extract, resulting in increased cell viability of only T47D cells, suggesting that increased ROS level is the underlying mechanism of cell death in T47D cells. Other antiproliferative studies employing *Euphorbia* species, such as *E. lathyrism* L., *E. antiquorum* L., and *E. fischeriana* Steud., have also found that increased ROS levels induced apoptosis [46,47,48]. Our results also revealed an immediate (2 s after treatment) release of calcium from the ER and mitochondrial calcium overload (27 s after treatment) in T47D cells, indicating that increased ROS levels trigger the accumulation of calcium in mitochondria. Other studies also revealed crosstalk between ROS levels and calcium signaling in different diseases, including cardiac physiology, pathologies, and neurodegeneration [49,50]. Blocking TRPV1 using capsazepine and chelating calcium with BAPTA-AM led to increased cell viability of MDA-MB-231 cells, suggesting that the activation of TRPV1 by *E. bicolor* xylene extract is the primary mechanism responsible for cell death in that cell line. Consequently, activation of TRPV1 causes an excessive influx of calcium in the cells, ultimately leading to apoptosis in MDA-MB-231 cells. Therefore, a potential approach for developing new drug therapies for triple-negative breast cancer could involve channel activators to induce calcium influx and cell death [16]. In T47D cells, blocking TRPV1 with CAPZ inhibited the effect of *E. bicolor* xylene extracts but only at small concentrations of extract (2–16 µg/mL), suggesting that TRPV1 may still be involved in reducing cell viability.

Experimental evidence indicates that when calcium homeostasis is disrupted, it leads to the breakdown of the mitochondrial membrane potential. This disruption also increases ROS levels and the release of proapoptotic proteins, resulting in apoptosis [13,51,52,53]. Mitochondrial Ca^2+^ overload was observed after *E. bicolor* xylene extract treatments of both T47D and MDA-MB-231 cells in our study (Figure 6), and therefore, we pursued the examination of apoptotic protein markers expression for both the mitochondrial intrinsic and extrinsic apoptotic pathways. It is known that mitochondrial depolarization releases cytochrome c into the cytoplasm, activating caspase-3 via caspase-9 [54]. Activation of caspase-3 is a well-recognized hallmark of apoptosis induced by other *Euphorbia* plant extracts such as *E. tirucalli* and *E. esula* L. [9,11,55,56,57]. *E. bicolor* xylene extract activated downstream caspases-3/9 (Figure 7), indicating the activation of the apoptotic pathways, as presented in the proposed models of apoptotic mechanisms in Figure 10 and Figure 11. Anti-apoptotic BCL-2 protein protects cancer cells by preventing apoptosis [58]. We observed reduced expression of BCL-2 protein in *E. bicolor* extract-treated T47D and MDA-MB-231 cells, suggesting that *E. bicolor* constituents of the xylene extract could be excellent candidates for targeting BCL-2 proteins in different cancers. Our results indicate that the BAX protein is expressed at a high molecular weight on Western blot. Studies indicated that BAX forms oligomers, necessary for its proapoptotic activity. The high molecular weight BAX oligomers bind to the mitochondrial membrane, leading to the mitochondrial intrinsic apoptotic pathway [16,59].

The xylene extract of *E. bicolor* activated the extrinsic apoptotic pathway, as evidenced by the expression of FAS and caspase-8 proteins (Figure 7, Figure 10 and Figure 11). These findings are supported by previous studies, which further suggest that the TRPV1 N-terminus can interact with proapoptotic FAS-associated proteins to trigger extrinsic apoptotic pathways [16,60,61]. It has been shown that TRPV1 is part of the ER membrane structure [62]. Its activation can lead to calcium disruption and cause ER stress [53], which can be an active source of calcium release in the cytoplasm. ER stress and compromised mitochondria can subsequently release apoptotic signals [16,54]. Our results show that *E. bicolor* xylene extract induced ER stress and ultimately triggered apoptosis in MDA-MB-231 breast cancer cells. In T47D cells, increased ROS levels may trigger ER stress and activate mitochondrial apoptosis as a result of *E. bicolor* xylene extract treatment. Expression of XBP1s, an activated (by splicing) transcription factor that facilitates ER stress [63], was observed in both *E. bicolor* extract treated T47D and MDA-MB-231 cells as an indication of ER-mediated stress, which stimulated the expression of CHOP, a transcription factor that increases the expression of other apoptotic factors [64] (Figure 9, Figure 10 and Figure 11).

Our results also suggest that *E. bicolor* xylene extract downregulates the PI3K/AKT signal transduction pathway (Figure 8, Figure 10 and Figure 11). The serine/threonine kinase AKT, also referred to as protein kinase B (PKB), is a key regulator of numerous cellular processes, including survival, growth, proliferation, cell cycle progression, and metabolism. Proper regulation of AKT activity is essential, since the balance between loss or gain of AKT activation contributes significantly to the pathophysiology of cancer [65,66]. We observed significantly low total and phosphorylated AKT and PI3K protein expressions in both *E. bicolor* xylene extract-treated T47D and MDA-MB-231 cells (Figure 8). It has been shown that PI3K/AKT pathway activation contributes to tumorigenesis, and inhibition of PI3K and AKT can decrease cellular proliferation and increase cell death [53]. Therefore, our results suggest that *E. bicolor* diterpenes could become potential candidates for targeting the PI3K/AKT signaling pathways in breast cancers.

The current study revealed two different antiproliferative mechanisms for the two breast cancer types, ER-positive and triple-negative, which most likely are because of their distinct molecular profiles. T47D cancer cells express estrogen and progesterone hormone receptors, the primary targets for current hormone therapies. MDA-MB-231 cancer cells lack hormone receptors, and therefore, they are not responsive to hormone therapies [67,68]. It is known that natural products have multiple molecular targets [69]. *E. bicolor* xylene extract may affect different targets depending on the cancer cells’ molecular characteristics, resulting in distinct antiproliferative molecular mechanisms.

In conclusion, our study presents for the first time the ROS-mediated antiproliferative mechanisms of action of *E. bicolor* xylene extract in T47D cells (Figure 10) and TRPV1-dependent mechanisms of action in MDA-MB-231 cells (Figure 11). *E. bicolor* xylene extract generates high ROS levels in T47D cells and triggers several apoptotic pathways. In MDA-MB-231 cells, *E. bicolor* xylene extract activates TRPV1 and induces mitochondrial and ER stress-mediated apoptotic pathways. In addition, the *E. bicolor* extract downregulates the PI3K/AKT signaling pathway in both T47D and MDA-MB-231 cells. Our findings suggest that *E. bicolor* xylene extract could be used to design specific therapeutics for cancer cell types. For ER-positive cell types, therapeutic agents could target the ROS-inducing apoptotic mechanism. For MDA-MB-231 cell types, the therapeutic mechanism could target the TRPV1-dependent apoptotic pathways.

## 4. Materials and Methods

### 4.1. Plant Extracts

*E. bicolor* plants were collected from fields around Denton, TX, USA, during September and October 2023 and a voucher specimen has been deposited in the TWU Herbarium. The ethanol extraction of aerial plant tissues (50 g) followed the procedure we employed before in our laboratory [32]. Xylene, a nonpolar solvent, was used to extract hydrophobic plant chemicals, especially diterpenes, following the protocol from Tidgewell (2007) with slight modifications [70]. Plant aerial tissues (50 g) were extracted in 200 proof xylene (Fisher Scientific, Newington, NH, USA) (1:4 *w*/*v*) at room temperature for one day. Filtered supernatants were placed in pre-weighed vials and dried under nitrogen [16]. Dried extract was dissolved in dimethyl sulfoxide (DMSO) (Fisher Scientific, Newington, NH, USA) in a 1:1 ratio and stored at −20 °C until use. The extract collected using ethanol solvent is named ethanol extract, and the extract collected using xylene solvent is named xylene extract. The ethanol extract represents a crude extract containing multiple classes of phytochemicals identified in our previous studies [29,32], whereas the xylene extract contains hydrophobic constituents. The presence of diterpenes in this fraction was qualitatively confirmed by TLC using resiniferatoxin and abietic acid as reference standards (Supplementary Materials [71,72,73,74]).

### 4.2. Cell Lines, Cell Cultures, and Treatments

The breast cancer cell lines under study, and adult human mammary epithelial cells (HMECs) were obtained from American Type Culture Collection (ATCC, Manassas, VA, USA). The cell culture media and conditions were as previously reported [16,32].

After 24 h, cells seeded into 96-well cell culture plates at 10,000 cells/well in a phenol-red-free medium were treated with increasing concentrations of *E. bicolor* ethanol, xylene extracts in DMSO (2, 4, 8, 16, 62.5, 125, 250, 500 µg/mL) or capsaicin at 0.5, 1, 5, 10, 50, 100, 250, and 500 µM concentrations. The final concentration of DMSO was <0.1%. Due to the limited availability and high cost of RTX, we chose to use capsaicin as a positive control for TRPV1 activation, a well-characterized TRPV1 activator and agonist of resiniferatoxin. This approach allowed us to reliably validate pathway activation while maintaining experimental consistency.

### 4.3. Antiproliferative Assays and IC50 Estimation

Antiproliferative activity of cells treated with increasing concentrations of *E. bicolor* ethanol, xylene extracts, or capsaicin was evaluated by performing MTS assays (Abcam, Waltham, MA, USA) as described before [16,32]. At least three separate experiments, each containing three replicates, were performed. HMECs showed reduction in cell viability starting at 125 µg/mL concentration using *E. bicolor* xylene extract. Therefore, the rest of the experiments were set up to use 62.5 µg/mL concentration to determine the antiproliferative mechanisms. The 50% inhibitory concentration (IC50) was estimated using the GraphPad Prism 9.4 software as described before [16,32].

### 4.4. Observations of Cytotoxic Effects

The IncuCyte live cell imaging system (Sartorius, Ann Arbor, MI, USA) was used to observe and capture photos at 4 h intervals of morphological changes of treated cells for several days. Cells were seeded into a 96-well plate (10,000 cells/well) and exposed to 62.5 µg/mL of *E. bicolor* xylene extract, before being placed into the IncuCyte [16].

### 4.5. TUNEL Assays

Cell apoptosis was assessed with the Click-iT™ Plus TUNEL assay kit (ThermoFisher Scientific, Waltham, MA, USA) as reported previously [16]. Cells were treated with *E. bicolor* xylene extract at a concentration of 62.5 µg/mL for 24 h before proceeding with the TUNEL assays.

### 4.6. Blocking TRPV1 with Capsazepine

Cells were pretreated with 10 µM of the TRPV1 antagonist capsazepine (Abcam, USA) for 30 min as previously described [16] before being treated with *E. bicolor* xylene extract (62.5 µg/mL) and cell viability measured using MTS assays. Three independent experiments were conducted.

### 4.7. Calcium Chelation

Cells were pretreated with 1 µM of the calcium chelator BAPTA-AM (Abcam, San Francisco, CA, USA), as previously described [16], and then treated with *E. bicolor* xylene extract (62.5 µg/mL) before MTS assays for cell viability were performed. Three independent experiments were conducted.

### 4.8. Visualization of TRPV1 Activation

The cytoplasmic calcium dynamics as the result of TRPV1 activation were observed using Fura2-AM staining. T47D and MDA-MB-231 cells were seeded into a 6-well plate and cultured overnight. After 24 h, cells were loaded with 5 μM of Fura2-AM for 30 min [14]. Following *E. bicolor* xylene treatment, the cytosolic Ca^2+^ signal was monitored continuously for 20 min using a LionHeart FX microscope (Agilent, Santa Clara, CA, USA).

### 4.9. ROS Detection

The intracellular ROS levels were determined using cell-permeant reagent 2’,7’-dichlorofluorescin diacetate (DCFDA) according to the manufacturer’s instructions (Abcam). Tert-butyl hydroperoxide (TBHP; 250 μM) was used as positive control. The fluorescence intensity values at each time point were calculated as the ratio of the value at a specific t-time point to the value at point zero time (t-time point/t0); t0 = first measurement. Briefly, cells were plated in FBS-supplemented medium without phenol red onto 96-well plates. After 24 h, the cells were washed once with 1× buffer provided in the kit, then the cells were incubated with 10 μM of DCFDA for 30 min at 37 °C, protected from light. Following incubation, the wells were washed with PBS, and treated with *E. bicolor* xylene extract. ROS production was determined immediately by measuring the formation of fluorescent dichlorofluorescein (DCF), using a Synergy microplate reader (Agilent, Winooski, VT, USA), at 485 nm excitation and 535 nm emission. Measurements were taken every 60 min for six hours. Three independent experiments were conducted.

### 4.10. N-acetyl-L-cysteine (NAC) Treatments to Block ROS Generation

Cells were pretreated with NAC (5 mM) for 1 h followed by treatment with or without *E. bicolor* xylene extract for another 72 h, and cell viability was measured using MTS assay. Three independent experiments were conducted.

### 4.11. Visualization of ER Calcium

ER-targeted low-affinity GCaMP6-210 variant, a fluorescent reporter for ER calcium signaling, was used to observe endoplasmic reticulum (ER) Ca^2+^ dynamics. T47D cells were seeded into a 24-well plate at 10.4 × 10^4^ cells in 500 µL phenol red free growth medium. After 24 h, cells were transfected with the GCaMP6-210 variant using Invitrogen™ Lipofectamine™ 3000 Transfection Reagent (Thermofisher Scientific, Waltham, MA, USA) according to the manufacturer’s instructions. Twelve hours after incubation, immediately after *E. bicolor* xylene extract treatment (62.5 µg/mL), calcium influx was observed with the LionHeart FX microscope.

### 4.12. Visualization of Mitochondrial Calcium

The mitochondrial calcium dynamics were observed using Rhod2-AM (Abcam, USA) as previously described [16]. Cells simultaneously loaded with 5 μM of Rhod2-AM, and 10 μM of MitoTracker Green (ThermoFisher Scientific, Waltham, MA, USA) were treated with *E. bicolor* xylene extracts (62.5 µg/mL) and fluorescence signals were monitored for 10 min with the LionHeart FX microscope.

### 4.13. Caspase 3 Activation Assay

Cells treated with *E. bicolor* xylene extract (62.5 µg/mL) for 24 h and incubated at 37 °C for 24 h. Detection of caspase 3 activation with the CellEvent™ Caspase-3/7 Green ReadyProbes™ reagent (ThermoFisher Scientific, Waltham, MA, USA) was performed as described before [16].

### 4.14. Western Blotting

As previously reported [16], Western blotting was carried out with a few changes. Proapoptotic and anti-apoptotic proteins were examined using total cellular protein. Preparation of cell lysates, estimation of total protein concentrations, SDS-PAGE, transfers onto polyvinylidene difluoride (PVDF) membranes (BioRad, Hercules, CA, USA), and probing with antibodies follow the procedure described before [16].

Antibodies used were as follows: anti-beta actin, anti-caspase 9, anti-caspase 8, anti-CHOP, anti-ATF4, anti-FAS, anti-PERK (mouse monoclonal antibody conjugated with HRP; 1:1000, *v*/*v*) (Santa Cruz Biotechnology, Dallas, TX, USA), anti-AKT, anti-pAKT (mouse monoclonal antibody conjugated with Alexa 488; 1:1000, *v*/*v*) (Santa Cruz Biotechnology, USA). Membranes were washed with Tris-buffered saline containing 0.1% (*v*/*v*) Tween 20 (TBST), incubated with enhanced chemiluminescence substrate solution (BioRad, USA), according to the manufacturer’s instructions, and visualized with a ChemiDoc system. For anti-Bcl-2, anti-BAX (rabbit monoclonal antibody; 1:1000, *v*/*v*) (Abcam, USA), anti-pPERK, anti-PI3K, anti-XBP1s (rabbit monoclonal antibody; 1:1000, *v*/*v*) (Cell Signaling Technology, Danvers, MA, USA), following the overnight incubation, the membranes were incubated with the secondary antibody (Goat Anti-Rabbit IgG H&L, Alexa Fluor^®^ 488; 1:500 *v*/*v*) (Thermofisher scientific, Waltham, MA, USA) for five minutes each after being rinsed three times in Tris-buffered saline containing 0.1% (*v*/*v*) Tween 20 (TBST) for 1 h.

### 4.15. Statistical Analyses

Means and standard errors of at least three experiments were calculated. One-way ANOVA was performed, followed by Tukey’s post hoc test to determine significant differences among the means for the antiproliferative (MTS) assays and fold expression analysis of Western blot (to compare DMSO control and *E. bicolor* xylene extract treatment) using GraphPad Prism 9.4. A *p*-value < 0.05 was considered statistically significant. Unpaired Welch’s *t*-test, to compare treated to DMSO control set, was used for TUNEL assays. A two-way ANOVA was performed, followed by the Dunnett test to determine significant differences among the means of Western blot fold expression (to compare DMSO control, *E. bicolor* xylene extract, and capsaicin treatment). ImageJ 1.5p software, (https://imagej.net/ij/download.html, accessed on 11 November 2023) was used to determine fold protein expression on Western blots.

## 5. Conclusions

Our study presents for the first time the ROS-mediated antiproliferative activity of *E. bicolor* xylene extract in T47D cells and TRPV1-dependent antiproliferative activity in MDA-MB-231 cells and their mechanisms of action. *E. bicolor* xylene extract generates high ROS levels in T47D cells and triggers several apoptotic pathways. In contrast, *E. bicolor* xylene extract activates TRPV1 and induces mitochondrial and endoplasmic reticulum stress-mediated apoptotic pathways in MDA-MB-231 cells. In addition, *E. bicolor* xylene extract downregulates the PI3K/AKT signaling pathway in both T47D and MDA-MB-231 cells. Our findings suggest that *E. bicolor* biochemicals could be used to design cancer cell type-specific therapeutics.

## Data Availability

Data are contained within the article. We did not use AI tools for the research process.

## Supplementary Materials

The following supporting information can be downloaded at: https://www.mdpi.com/article/10.3390/ijms27020962/s1.

## References

[1] Kim J., Harper A., McCormack V., Sung H., Houssami N., Morgan E., Mutebi M., Garvey G., Soerjomataram I., Fidler-Benaoudia M. (2025). Global patterns and trends in breast cancer incidence and mortality across 185 countries. Nat. Med..

[2] Giaquinto A.N., Sung H., Miller K.D., Kramer J.L., Newman L.A., Minihan A., Jemal A., Siegel R.L. (2022). Breast cancer statistics, 2022. CA Cancer J. Clin..

[3] Siegel R.L., Miller K.D., Wagle N.S., Jemal A. (2023). Cancer statistics, 2023. CA Cancer J. Clin..

[4] Chikara S., Nagaprashantha L.D., Singhal J., Horne D., Awasthi S., Singhal S.S. (2018). Oxidative stress and dietary phytochemicals: Role in cancer chemoprevention and treatment. Cancer Lett..

[5] Pucci C., Martinelli C., Ciofani G. (2019). Innovative approaches for cancer treatment: Current perspectives and new challenges. Ecancermedicalscience.

[6] Liu Y.-Q., Wang X.-L., He D.-H., Cheng Y.-X. (2021). Protection against chemotherapy- and radiotherapy-induced side effects: A review based on the mechanisms and therapeutic opportunities of phytochemicals. Phytomedicine.

[7] de Carvalho A.P.A., Conte-Junior C.A. (2021). Health benefits of phytochemicals from Brazilian native foods and plants: Antioxidant, antimicrobial, anti-cancer, and risk factors of metabolic/endocrine disorders control. Trends Food Sci. Technol..

[8] Alibakhshi A., Malekzadeh R., Hosseini S.A., Yaghoobi H. (2023). Investigation of the therapeutic role of native plant compounds against colorectal cancer based on system biology and virtual screening. Sci. Rep..

[9] Khan M.I., Bouyahya A., Hachlafi N.E., Menyiy N.E., Akram M., Sultana S., Zengin G., Ponomareva L., Shariati M.A., Ojo O.A. (2022). Anticancer properties of medicinal plants and their bioactive compounds against breast cancer: A review on recent investigations. Environ. Sci. Pollut. Res..

[10] Shrihastini V., Muthuramalingam P., Adarshan S., Sujitha M., Chen J.-T., Shin H., Ramesh M. (2021). Plant-derived bioactive compounds, their anti-cancer effects, and in silico approaches as an alternative target treatment strategy for breast cancer: An updated overview. Cancers.

[11] Khan T., Ali M., Khan A., Nisar P., Jan S.A., Afridi S., Shinwari Z.K. (2019). Anticancer plants: A review of active phytochemicals, applications in animal models, and regulatory aspects. Biomolecules.

[12] Tomiotto-Pellissier F., Gonçalves M.D., Silva T.F., Concato V.M., da Silva Bortoleti B.T., Arakawa N.S., Conchon-Costa I., Pavanelli W.R., Panis C. (2022). Plant-derived diterpenes for breast cancer treatment: New perspectives and recent advances. Stud. Nat. Prod. Chem..

[13] Gao X., Li X., Ho C.-T., Lin X., Zhang Y., Li B., Chen Z. (2020). Cocoa tea (*Camellia ptilophylla*) induces mitochondria-dependent apoptosis in HCT116 cells via ROS generation and PI3K/Akt signaling pathway. Food Res. Int..

[14] Xu S., Cheng X., Wu L., Zheng J., Wang X., Wu J., Yu H., Bao J., Zhang L. (2020). Capsaicin induces mitochondrial dysfunction and apoptosis in anaplastic thyroid carcinoma cells via TRPV1-mediated mitochondrial calcium overload. Cell. Signal..

[15] Tajada S., Villalobos C. (2020). Calcium permeable channels in cancer hallmarks. Front. Pharmacol..

[16] Rumpa M.M., Maier C. (2024). TRPV1-dependent antiproliferative activity of *Maclura pomifera* extracts in estrogen receptor positive breast cancer involves multiple apoptotic pathways. Int. J. Mol. Sci..

[17] Calixto J.B., Kassuya C.A., André E., Ferreira J. (2005). Contribution of natural products to the discovery of the transient receptor potential (TRP) channels family and their functions. Pharmacol. Ther..

[18] Zhang M., Ma Y., Ye X., Zhang N., Pan L., Wang B. (2023). TRP (transient receptor potential) ion channel family: Structures, biological functions and therapeutic interventions for diseases. Signal Transduct. Target. Ther..

[19] Rosenberger D.C., Binzen U., Treede R.-D., Greffrath W. (2020). The capsaicin receptor TRPV1 is the first line defense protecting from acute non damaging heat: A translational approach. J. Transl. Med..

[20] Asadi-Samani M., Rafieian-Kopaei M., Lorigooini Z., Shirzad H. (2019). The effect of *Euphorbia szovitsii* Fisch. & CAMey extract on the viability and the proliferation of MDA-MB-231 cell line. Biosci. Rep..

[21] Bao Z., Dai X., Wang P., Tao Y., Chai D. (2019). Capsaicin induces cytotoxicity in human osteosarcoma MG63 cells through TRPV1-dependent and-independent pathways. Cell Cycle.

[22] Nie R., Liu Q., Wang X. (2022). TRPV1 is a potential tumor suppressor for its negative association with tumor proliferation and positive association with antitumor immune responses in pan-cancer. J. Oncol..

[23] Weber L.V., Al-Refae K., Wölk G., Bonatz G., Altmüller J., Becker C., Gisselmann G., Hatt H. (2016). Expression and functionality of TRPV1 in breast cancer cells. Breast Cancer Targets Ther..

[24] Zhai K., Liskova A., Kubatka P., Büsselberg D. (2020). Calcium entry through TRPV1: A potential target for the regulation of proliferation and apoptosis in cancerous and healthy cells. Int. J. Mol. Sci..

[25] Bujak J.K., Kosmala D., Szopa I.M., Majchrzak K., Bednarczyk P. (2019). Inflammation, cancer and immunity-implication of TRPV1 channel. Front. Oncol..

[26] Nogueira M.L., Fava W.S., Correia J.B., Araújo R.V.D., Martins R.C., Dos Santos A.B.C., Filho F.J.C.M., Da Costa R.B. (2024). Medicinal species of the genus croton (*Euphorbiaceae*): A worldwide view on the dynamics and evolution of scientific production. Rev. Gest. Soc. Ambient..

[27] Zhao H., Sun L., Kong C., Mei W., Dai H., Xu F., Huang S. (2022). Phytochemical and pharmacological review of diterpenoids from the genus *Euphorbia Linn* (2012–2021). J. Ethnopharmacol..

[28] Radi M.H., El-Shiekh R.A., El-Halawany A.M., Al-Abd A.M., Abdel-Sattar E. (2023). In Vitro cytotoxic study of *Euphorbia grantii* Oliv. aerial parts against MCF-7 and MCF-7ADR breast cancer cell lines: A bioactivity-guided isolation. ACS Omega.

[29] Basu P., Tongkhuya S.A., Harris T.L., Riley A.R., Maier C., Granger J., Wojtaszek J., Averitt D.L. (2019). *Euphorbia bicolor* (*Euphorbiaceae*) latex phytochemicals induce long-lasting non-opioid peripheral analgesia in a rat model of inflammatory pain. Front. Pharmacol..

[30] Benjamaa R., Moujanni A., Kaushik N., Choi E.H., Essamadi A.K., Kaushik N.K. (2022). Euphorbia species latex: A comprehensive review on phytochemistry and biological activities. Front. Plant Sci..

[31] Yang Y., Chen X., Luan F., Wang M., Wang Z., Wang J., He X. (2021). *Euphorbia helioscopia* L.: A phytochemical and pharmacological overview. Phytochemistry.

[32] Basu P., Meza E., Bergel M., Maier C. (2020). Estrogenic, antiestrogenic and antiproliferative activities of *Euphorbia bicolor* (*Euphorbiaceae*) latex extracts and its phytochemicals. Nutrients.

[33] Sadeghi-Aliabadi H., Sajjadi S.E., Khodamoradi M. (2009). Cytotoxicity of *Euphorbia macroclada* on MDA-MB-468 breast cancer cell line. Iran. J. Pharm. Sci..

[34] Elokely K., Velisetty P., Delemotte L., Palovcak E., Klein M.L., Rohacs T., Carnevale V. (2016). Understanding TRPV1 activation by ligands: Insights from the binding modes of capsaicin and resiniferatoxin. Proc. Natl. Acad. Sci. USA.

[35] Farfariello V., Liberati S., Morelli M.B., Tomassoni D., Santoni M., Nabissi M., Giannantoni A., Santoni G., Amantini C. (2014). Resiniferatoxin induces death of bladder cancer cells associated with mitochondrial dysfunction and reduces tumor growth in a xenograft mouse model. Chem. Biol. Interact..

[36] Hua H., Zhang H., Chen J., Wang J., Liu J., Jiang Y. (2021). Targeting Akt in cancer for precision therapy. J. Hematol. Oncol..

[37] Chiang J.-H., Tsai F.-J., Hsu Y.-M., Yin M.-C., Chiu H.-Y., Yang J.-S. (2020). Sensitivity of allyl isothiocyanate to induce apoptosis via ER stress and the mitochondrial pathway upon ROS production in colorectal adenocarcinoma cells. Oncol. Rep..

[38] Arnold M., Morgan E., Rumgay H., Mafra A., Singh D., Laversanne M., Vignat J., Gralow J.R., Cardoso F., Siesling S. (2022). Current and future burden of breast cancer: Global statistics for 2020 and 2040. Breast.

[39] Siddiqui A.J., Jahan S., Singh R., Saxena J., Ashraf S.A., Khan A., Choudhary R.K., Balakrishnan S., Badraoui R., Bardakci F. (2022). Plants in anticancer drug discovery: From molecular mechanism to chemoprevention. Biomed Res. Int..

[40] Kwan Y.P., Saito T., Ibrahim D., Al-Hassan F.M.S., Oon C.E., Chen Y., Jothy S.L., Kanwar J.R., Sasidharan S. (2016). Evaluation of the cytotoxicity, cell-cycle arrest, and apoptotic induction by *Euphorbia hirta* in MCF-7 breast cancer cells. Pharm. Biol..

[41] Taş A., Şahin Bölükbaşı S., Çevik E., Ozmen E., Gümüş E., Siliğ Y. (2018). An in vitro study of cytotoxic activity of *Euphorbia macroclada* Boiss on MCF-7 cells. Indian J. Pharm. Educ. Res..

[42] Choene M., Motadi L. (2016). Validation of the antiproliferative effects of *Euphorbia tirucalli* extracts in breast cancer cell lines. Mol. Biol..

[43] Monteith G.R., McAndrew D., Faddy H.M., Roberts-Thomson S.J. (2007). Calcium and cancer: Targeting Ca^2+^ transport. Nat. Rev. Cancer.

[44] Varghese E., Samuel S.M., Sadiq Z., Kubatka P., Liskova A., Benacka J., Pazinka P., Kruzliak P., Büsselberg D. (2019). Anti-cancer agents in proliferation and cell death: The calcium connection. Int. J. Mol. Sci..

[45] Wu T.T., Peters A.A., Tan P.T., Roberts-Thomson S.J., Monteith G.R. (2014). Consequences of activating the calcium-permeable ion channel TRPV1 in breast cancer cells with regulated TRPV1 expression. Cell Calcium.

[46] Zhu A., Sun Y., Zhong Q., Yang J., Zhang T., Zhao J., Wang Q. (2019). Effect of euphorbia factor L1 on oxidative stress, apoptosis, and autophagy in human gastric epithelial cells. Phytomedicine.

[47] Hsieh W.-T., Lin H.-Y., Chen J.-H., Kuo Y.-H., Fan M.-J., Wu R.S.-C., Wu K.-C., Wood W.G., Chung J.-G. (2011). Latex of *Euphorbia antiquorum* induces apoptosis in human cervical cancer cells via c-jun n-terminal kinase activation and reactive oxygen species production. Nutr. Cancer.

[48] Ma L., Chen Z., Li J., Zhang H., Jia Y., Liu J. (2021). DP from *Euphorbia fischeriana* S. mediated apoptosis in leukemia cells via the PI3k/Akt signaling pathways. J. Ethnopharmacol..

[49] Baev A.Y., Vinokurov A.Y., Novikova I.N., Dremin V.V., Potapova E.V., Abramov A.Y. (2022). Interaction of mitochondrial calcium and ROS in neurodegeneration. Cells.

[50] De Nicolo B., Cataldi-Stagetti E., Diquigiovanni C., Bonora E. (2023). Calcium and reactive oxygen species signaling interplays in cardiac physiology and pathologies. Antioxidants.

[51] Rajendran P., Maheshwari U., Muthukrishnan A., Muthuswamy R., Anand K., Ravindran B., Dhanaraj P., Balamuralikrishnan B., Chang S.W., Chung W.J. (2021). Myricetin: Versatile plant based flavonoid for cancer treatment by inducing cell cycle arrest and ROS–reliant mitochondria-facilitated apoptosis in A549 lung cancer cells and in silico prediction. Mol. Cell. Biochem..

[52] Zhao X., Tao X., Xu L., Yin L., Qi Y., Xu Y., Han X., Peng J. (2016). Dioscin induces apoptosis in human cervical carcinoma HeLa and SiHa cells through ROS-mediated DNA damage and the mitochondrial signaling pathway. Molecules.

[53] Almilaibary A. (2024). Phyto-therapeutics as anti-cancer agents in breast cancer: Pathway targeting and mechanistic elucidation. Saudi J. Biol. Sci..

[54] Marchi S., Patergnani S., Missiroli S., Morciano G., Rimessi A., Wieckowski M.R., Giorgi C., Pinton P. (2018). Mitochondrial and endoplasmic reticulum calcium homeostasis and cell death. Cell Calcium.

[55] Alkhalaf M., El-Mowafy A., Renno W., Rachid O., Ali A., Al-Attyiah R. (2008). Resveratrol-induced apoptosis in human breast cancer cells is mediated primarily through the caspase-3-dependent pathway. Arch. Med. Res..

[56] Archanjo A.B., de Paula Careta F., Costa A.V., Nunes L.D.C. (2016). Evaluation of cytotoxicity and expression of caspase-3 and p53 in HCT-116 cells of lineage treated with different extracts of *Euphorbia tirucalli* L. Arch. Vet. Sci..

[57] Fu Z.-Y., Han X.-D., Wang A.-H., Liu X.-B. (2016). Apoptosis of human gastric carcinoma cells induced by *Euphorbia esula* latex. World J. Gastroenterol..

[58] Basu A. (2022). The interplay between apoptosis and cellular senescence: Bcl-2 family proteins as targets for cancer therapy. Pharmacol. Ther..

[59] Antonsson B., Montessuit S., Sanchez B., Martinou J.-C. (2001). Bax is present as a high molecular weight oligomer/complex in the mitochondrial membrane of apoptotic cells. J. Biol. Chem..

[60] Kim S., Kang C., Shin C.Y., Hwang S.W., Yang Y.D., Shim W.S., Park M.-Y., Kim E., Kim M., Kim B.-M. (2006). TRPV1 recapitulates native capsaicin receptor in sensory neurons in association with Fas-associated factor 1. J. Neurosci..

[61] Abd El-Hafeez A.A., Marzouk H.M.M., Abdelhamid M.A., Khalifa H.O., Hasanin T.H.A., Habib A.G.K., Abdelwahed F.M., Barakat F.M., Bastawy E.M., Abdelghani E.M.B. (2022). Anti-cancer effect of *Hyoscyamus muticus* extract via its activation of Fas/FasL-ASK1-p38 pathway. Biotechnol. Bioprocess Eng..

[62] Zhao R., Tsang S.Y. (2017). Versatile roles of intracellularly located TRPV1 channel. J. Cell. Physiol..

[63] Park S.-M., Kang T.-I., So J.-S. (2021). Roles of XBP1s in transcriptional regulation of target genes. Biomedicines.

[64] Hu H., Tian M., Ding C., Yu S. (2019). The C/EBP homologous protein (CHOP) transcription factor functions in endoplasmic reticulum stress-induced apoptosis and microbial infection. Front. Immunol..

[65] He Y., Sun M.M., Zhang G.G., Yang J., Chen K.S., Xu W.W., Li B. (2021). Targeting PI3K/Akt signal transduction for cancer therapy. Signal Transduct. Target. Ther..

[66] Yang J., Nie J., Ma X., Wei Y., Peng Y., Wei X. (2019). Targeting PI3K in cancer: Mechanisms and advances in clinical trials. Mol. Cancer.

[67] Bou Zerdan M., Ghorayeb T., Saliba F., Allam S., Zerdan M.B., Yaghi M., Bilani N., Jaafar R., Nahleh Z. (2022). Triple negative breast cancer: Updates on classification and treatment in 2021. Cancers.

[68] Beňačka R., Szabóová D., Guľašová Z., Hertelyová Z., Radoňák J. (2022). Classic and new markers in diagnostics and classification of breast cancer. Cancers.

[69] Chen X., Li H. (2024). Bruceine D and Narclasine inhibit the proliferation of breast cancer cells and the prediction of potential drug targets. PLoS ONE.

[70] Tidgewell K.J. (2007). Development of Novel Analgesics from the Neoclerodane Diterpene Natural Product Salvinorin A. Ph.D. Thesis.

[71] Hajdú Z., Hohmann J., Forgo P., Martinek T., Dervarics M., Zupkó I., Falkay G., Cossuta D., Máthé I. (2007). Diterpenoids and flavonoids from the fruits of *Vitex agnus-castus* and antioxidant activity of the fruit extracts and their constituents. Phytother. Res. Int. J. Devoted Pharmacol. Toxicol. Eval. Nat. Prod. Deriv..

[72] Mukadam S., Ghule C., Girme A., Shinde V.M., Hingorani L., Mahadik K.R. (2023). A simple HPTLC approach of quantification of serratol and tirucallic acid with boswellic acids in *Boswellia serrata* by validated densitometric method with MS/MS characterization. J. Chromatogr. Sci..

[73] Schlemmer W., Egger M., Dächert M., Lahti J., Gschiel M., Walzl A., Leitner E., Spirk S., Hirn U. (2020). Rapid separation and quantitative analysis of complex lipophilic wood pulp extractive mixtures based on 2D thin layer chromatography. ACS Sustain. Chem. Eng..

[74] Vasas A., Hohmann J. (2014). Euphorbia diterpenes: Isolation, structure, biological activity, and synthesis (2008–2012). Chem. Rev..

