# Supplementary material for: Euphorbia bicolor Xylene Extract Induces Mitochondrial and Endoplasmic Reticulum Stress-Mediated Apoptotic Pathways in MDA-MB-231 and T47D Cells
Source: Int J Mol Sci. 2026 Jan 18;27(2):962. doi: 10.3390/ijms27020962 (PMC12842104; doi:10.3390/ijms27020962)
Supplement: Supplementary file 1 [file ijms-27-00962-s001.zip › ijms-4072443-supplementary.pdf]

Supplementary Information
Thin-layer chromatography (TLC) analyses showing the presence of the diterpenes resiniferatoxin and abietic acid in E. bicolor xylene extract

Thin-layer chromatography (TLC) analyses were performed using silica gel 60 F_{254} TLC plates (Fisher scientific). Reference compounds, abietic acid and resiniferatoxin, were dissolved in ethanol. 10 µL samples of xylene and ethanol extracts were applied to the TLC plates and allowed to dry under ambient conditions prior to development.
Chromatographic separation for TLC plates 1 and 3 were carried out using an acidic solvent system consisting of hexane: ethyl acetate: methanol: formic acid (35:45:10:10, v/v/v/v). For TLC plate 2, a basic solvent system of hexane/toluene (90:10, v/v) was used. Plates were developed until the solvent front reached the top of the plate, after which they were removed from the developing chambers and air-dried. TLC plates 1 and 3 were visualized under UV light at 254 nm, and TLC plate 1 again visualized by exposure to iodine vapor for 5 min. TLC plate 2 was visualized by spraying with a sulfuric acid: methanol: glacial acetic acid (5:85:10, v/v/v), followed by heating at 110 °C for 15 minutes.

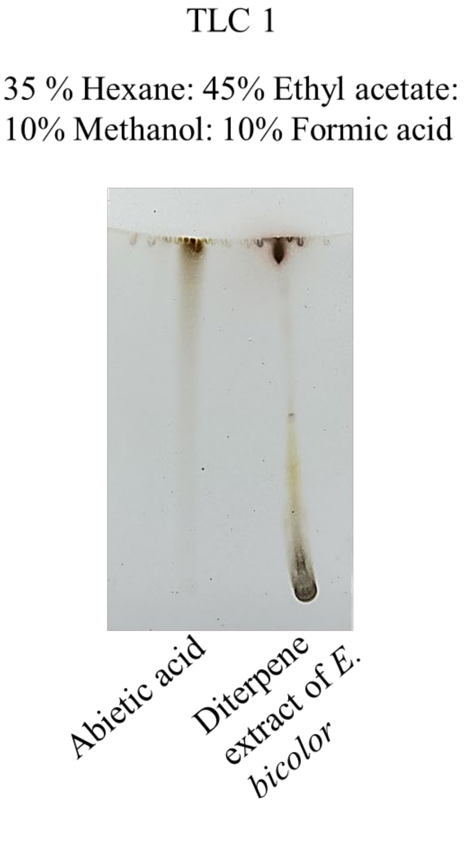

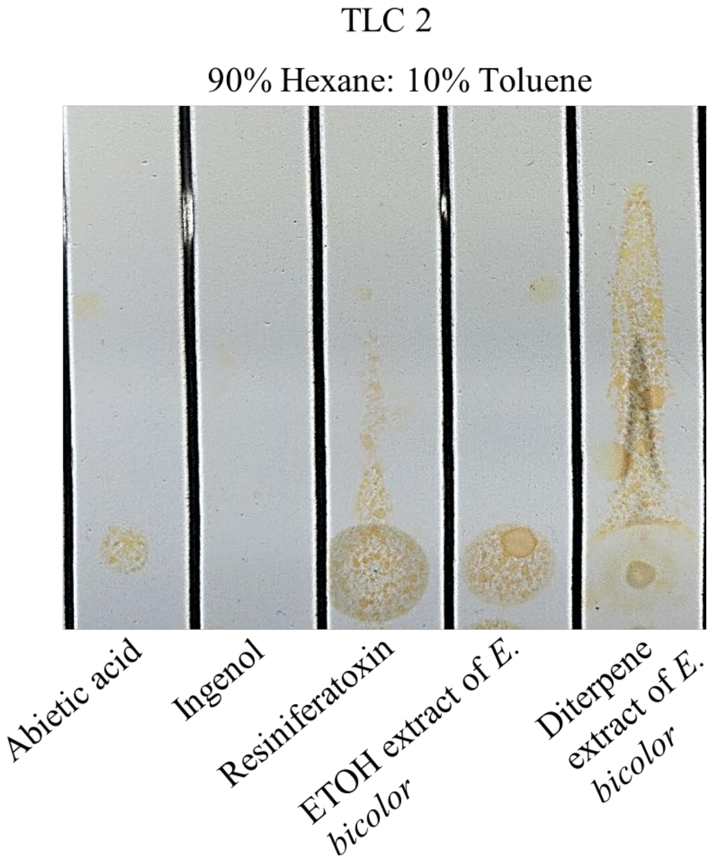

TLC 1, visualized by UV

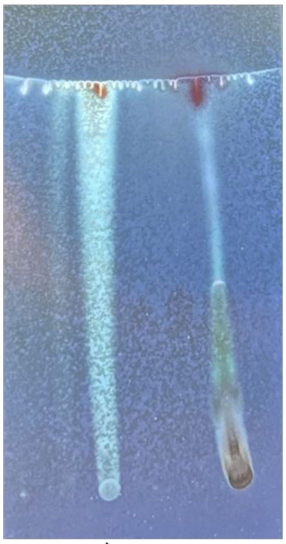

Abietic acid

Diterpene extract of *E. bicolor*

TLC 3

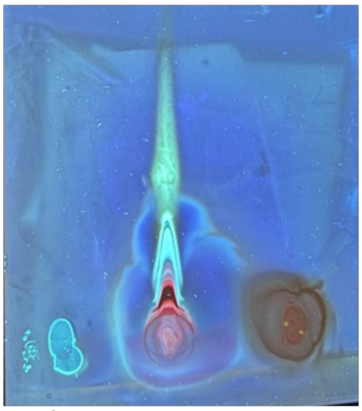

Resiniferatoxin

Diterpene extract of *E. bicolor*

ETOH extract of *E. bicolor*
